# HOW DOES MODE OF SERVICE DELIVERY IMPACT CAREGIVER STRAIN? A MIXED-METHODS STUDY OF CAREGIVERS IN LOS ANGELES

**DOI:** 10.1093/geroni/igz038.1233

**Published:** 2019-11-08

**Authors:** Haley B Gallo, Chia Ying Chen, Donna Benton

**Affiliations:** 1 Leonard Davis School of Gerontology, University of Southern California, Los Angeles, California, United States; 2 University of Southern California, Los Angeles, California, United States

## Abstract

As the number of people living with Alzheimer’s disease and related dementias increases, so too will the number of people who care for them. This growing population requires instrumental and emotional support as they fulfill their caregiving duties. CareJourney is an online platform that provides this support; it can be used either alone, or with traditional in-person and phone-mediated consultations with family care navigators (FCNs). The intent of CareJourney is to provide a greater opportunity for self-directed services, as well as flexibility for working caregivers and those who prefer using technology. Satisfaction surveys (N=222) were analyzed to evaluate caregivers’ perceptions of CareJourney and the traditional service delivery modes. Additionally, five 30-minute interviews with caregivers were coded by two researchers (kappa=0.84) for a more in-depth understanding. Ten percent of survey respondents used CareJourney, and preliminary results from both the interviews and the survey suggest that caregiver burden, lack of time, and desire for a personal connection with FCNs influence mode of service delivery. Although a quarter of users relied on CareJourney to find caregiving resources and email their FCNs because it was more convenient than traditional modes, the interviews and surveys revealed that the caregivers most valued the personal touch that came from interacting with FCNs. Speaking with FCNs over the phone or in-person allowed caregivers to feel “reassured,” “less isolated,” and “comforted” as they discussed caregiving strains. Future interventions and services for caregivers should be offered using multiple modes to accommodate a range of time-demands and preferences for personal connection.

